# High prevalence of resistance to third-generation cephalosporins detected among clinical isolates from sentinel healthcare facilities in Lagos, Nigeria

**DOI:** 10.1186/s13756-022-01171-2

**Published:** 2022-11-08

**Authors:** Emelda E. Chukwu, Oluwatoyin B. Awoderu, Christian A. Enwuru, Ebelechukwu E. Afocha, Rahman G. Lawal, Rahaman A. Ahmed, Ishola Olanrewaju, Chika K. Onwuamah, Rosemary A. Audu, Folasade T. Ogunsola

**Affiliations:** 1 Antimicrobial Resistance Research group, Nigerian Institute of Medical Research, Yaba, Lagos State Nigeria; 2 Center for Human Virology and Genomics, Microbiology Department, Nigerian Institute of Medical Research, Yaba, Lagos State Nigeria; 3 Department of Medical Microbiology and Parasitology, College of Medicine, University of Lagos, Idi-Araba, Nigeria

**Keywords:** Antibiotics, Antimicrobial resistance, *MecA*, Clinical isolates, Resistance genes, ESBL genes, *Staphylococcus aureus*, Nigeria

## Abstract

**Background:**

Antimicrobial resistance (AMR) in bacterial pathogens is a worldwide concern that demands immediate attention. Most information on AMR originates from high-income countries and little is known about the burden in Africa, particularly Nigeria. Using four sentinel sites (General hospitals) in Lagos State, this study sought to estimate the burden of AMR.

**Methods:**

This is a hospital-based surveillance using secondary health care centres. Four sites were randomly selected and included in the study. Clinical isolates were collected over a period of 6 months for each site from August 2020 to March 2021. All isolates were characterised and analysed for resistance to 15 antibiotics using the Kirby-Baur method. Multiplex PCR assay was used for the detection of Extended spectrum beta lactamase genes. Data analysis was done using SPSS version 27.0.

**Results:**

Four hundred and ninety-nine (499) patients consented and participated in this study, consisting of 412 (82.6%) females and 87 (17.4%) males. The mean age ± SD of the participants was 33.9 ± 13.8 with a range of 1–89 years. The majority (90.8%) of the participants were outpatients. Two hundred and thirty-two (232) isolates were obtained from 219 samples, comprising of 120 (51.7%) Gram positive and 112 (48.3%) Gram negative organisms. Key bacterial pathogens isolated from this study included *Staphylococcus aureus* (22.8%), *Escherichia coli* (16.4%), *Staphylococcus* spp. (15.9%), *Enterococcus* spp. (7.3%) and *Klebsiella pneumoniae* (6.5%). There was high prevalence of multi-drug resistance (79.3%) among the isolates with 73.6% of *Staphylococcus aureus* phenotypically resistant to methicillin and 70% possessed the *MecA* gene. 76.5% of *Enterococcus* spp. isolated were Vancomycin resistant. Overall, resistance to Cephalosporins was most frequently/commonly observed (Cefotaxime 87.5%).

**Conclusion:**

A high incidence of AMR was identified in clinical bacteria isolates from selected general hospitals in Lagos State, highlighting the necessity for the implementation of national action plans to limit the prevalence of AMR. Surveillance via collection of isolates has a lot of promise, especially in resource-limited environments.

**Supplementary Information:**

The online version contains supplementary material available at 10.1186/s13756-022-01171-2.

## Introduction

Antimicrobial resistance (AMR) in bacterial pathogens has been established as a worldwide threat requiring urgent attention. Africa, with its high infectious disease burden, is hampered by paucity of data on the burden of AMR. The role of antibiotics in the treatment of infectious illnesses cannot be overemphasized. However, the prevalence of infectious diseases associated with cases of multidrug-resistant (MDR) bacteria have been steadily rising [1].

AMR has reached epidemic magnitudes, increasing public health concerns because there are few novel antibiotics in development, especially for Gram-negative bacteria [2]. Therapeutic options for infections due to bacteria have become increasingly limited. Poor regulation of over-the-counter purchase of antibiotics remains an issue in Nigeria, more so in Lagos state with the number of patent and proprietary medication shops projected to be 1374 per 100,000 population in each local government area [3]. Many of these drug vendors and community pharmacies purchase and stock, controlled drugs including antibiotics that are outside their licensing and supply them over-the-counter without regulations [4].

The incidence of extended spectrum beta-lactamase (ESBL) producing pathogenic bacteria is increasing in Nigeria. In research carried out in 2010 in Kano, North-western Nigeria to test for ESBL production among Enterobacteriaceae isolates, an ESBL prevalence of 9.25% was identified [5]. Similarly, a study in 2010 conducted at a tertiary health institution in Ogun State, Southwestern Nigeria, to determine ESBL prevalence in *Escherichia coli* and *Klebsiella species*, reported an ESBL prevalence of 2.5% for *Escherichia coli* and 5% for *Klebsiella pneumoniae* [6]. A higher prevalence was reported in 2016 by Mohammed et al. [7] with an ESBL prevalence of 23.8% for *Escherichia coli* and 30.0% for *Klebsiella species* in a teaching hospital in North-eastern Nigeria. In a recent study, Akinyemi et al. [8] identified a significantly higher incidence of 69.8% among *Klebsiella pneumoniae* isolated from clinical samples from four medical centres in Lagos State. The problem of AMR can be exacerbated by misconceptions on the use of antibiotics leading to overuse and misuse. A recent survey revealed that many Nigerians believe that catarrh, cold and flu, and measles, are conditions requiring antibiotics [9].

To combat AMR, the first step proposed in the WHO’s Global Action Plan (GAP) is to conduct a baseline evaluation of AMR prevalence in all countries [10]. Leopold et al. [11] conducted a systematic review of AMR in Sub-Saharan Africa, highlighting the limitations of current data and revealing a significant incidence of AMR to routinely used antibiotics in clinical bacterial isolates. As a result, more effort needs to be made in a stepwise approach towards the implementation of a surveillance plan. Appropriate data pertaining to the epidemiology of AMR can be obtained via phenotypic and genotypic detection of AMR strains of public health importance. Surveillance data can help influence clinical therapy decisions and policy, and strategy formulation for AMR control. As a preliminary step toward monitoring trends of AMR development and spread in Lagos, this study provides information on the prevalence of AMR in bacterial clinical isolates in Lagos State.

## Methods

### Study design

This is a hospital-based survey which was piloted in selected General hospitals (secondary health care centres) in Lagos to assess the prevalence of AMR and ESBL production in bacteria strains from clinical samples. The sentinel sites were randomly selected because their combined catchment area covered most of the Lagos population and they also had appropriate/basic laboratory facilities and personnel for the detection of the bacteria pathogens of interest which was ensured using a checklist (Additional file 1). All bacterial isolates from clinical specimens in selected general hospitals were included. Only participants who gave their consent to provide clinical and laboratory data were enrolled for the study. Structured questionnaires were used to obtain demographic information as well as predictors for assessing the relative risk of AMR.

### The study location/site

**Fig. 1:**
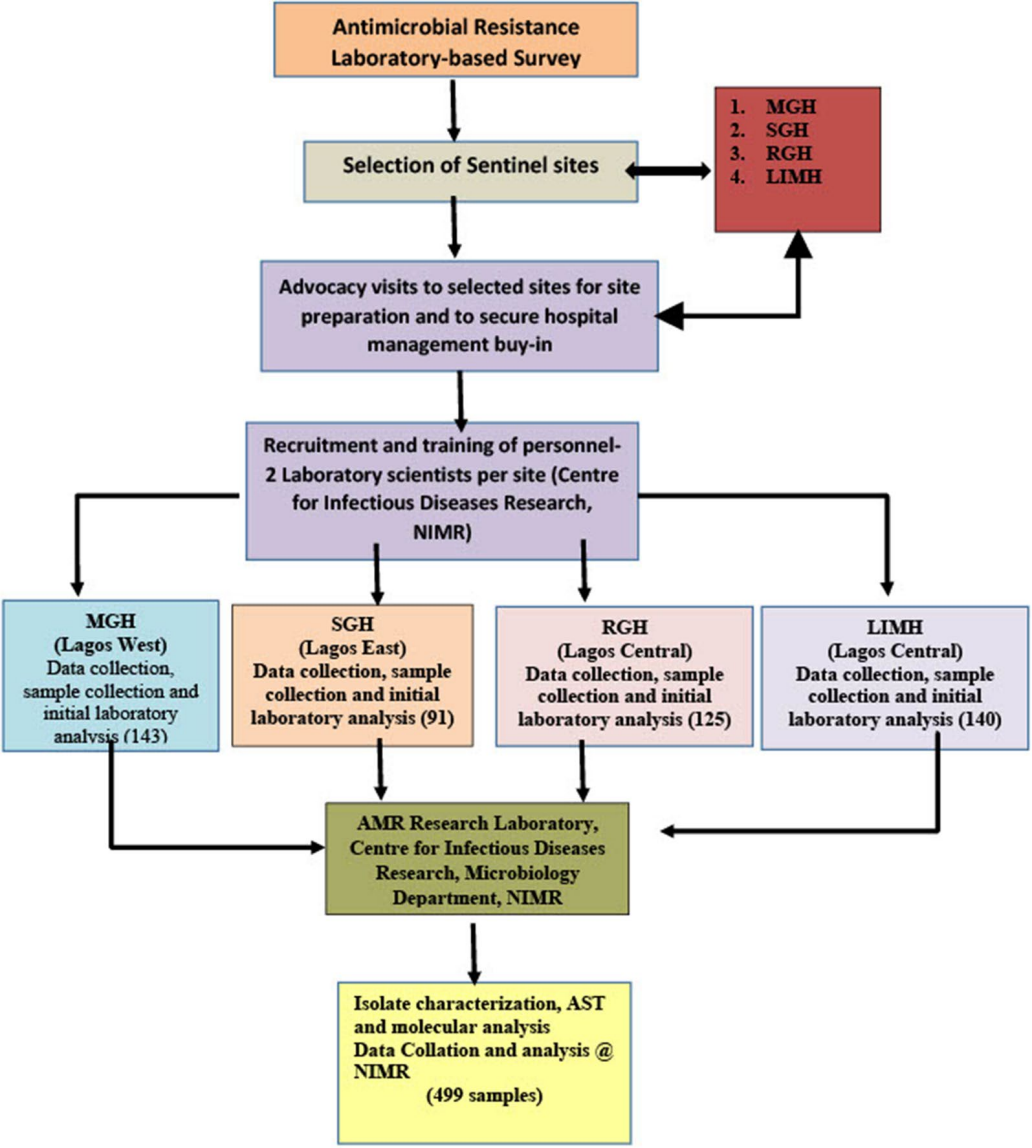
Schematic representation of Study design

The study was carried out in Lagos State. The metropolis of Lagos is a low-lying and densely populated coastal area in the southwestern part of Nigeria. The study sites were mapped based on the three senatorial districts in Lagos state (Lagos East, Lagos west and Lagos central). Stratified random sampling method was used to select two secondary health care facilities from each district giving a total of six General Hospitals. However, two of the facilities pulled out due to some logistical constraints which prevented them from meeting the requirements for the study. The four sites used for this study included Lagos Island Maternity Hospital (LIMH), Mushin General Hospital (MGH), Randle General Hospital (RGH) and Shomolu General Hospital (SGH), see Fig. 1.

### Recruitment and training of personnel at the collection centres

Two medical laboratory scientists (microbiology specialty) working at the Microbiology department of the hospital laboratory and the Head of the Department were selected from each center for a training workshop prior to commencing the study. Study tools and procedures were harmonized, and they were trained on questionnaire administration, appropriate sample collection, cultivation and isolation methods.

### Sample collection/ initial cultivation and isolation

Routine, clinical sample specimens including sputum, urine, stool and swabs from wounds, vagina, cervix, ear, eye and throat were collected at the sentinel sites (General Hospitals). All individuals who presented at the four participating centers during the study period and gave their consent to participate were included in the study. The samples were collected over a period of 6 months for each site from August 2020 to March 2021. All four centers completed 6 months of sample collection from their commencement date. Specimens were analysed following the routine protocol for bacteria isolation. Pure growth colonies were presumptively identified and stored. All bacteria strains were placed on agar slants and transferred to the Microbiology department of the Nigerian Institute of Medical Research where further microbiological and molecular analysis were carried out.

### Collection, characterization and storage of isolates

Isolates were characterized using routine biochemical methods. The identity was subsequently confirmed using BD BioMic V3 biochemical rating Identification system: —BioMic V3 is a semi-automated bacteriological identification system (Becton Dickinson, USA). Identified isolates were stored in duplicates at − 20 °C using 20% Glycerol-Brain heart infusion (BHI) broth and skimmed milk until required for further processing.

### Detection of strains of MRSA by cefoxitin disc diffusion method

Susceptibility of *Staphylococcus aureus* isolates to cefoxitin (30 µg) was determined by modified Kirby-Bauer disc diffusion method following Clinical Laboratory Standard Institute (CLSI) guidelines [12] to screen for methicillin resistance. All strains of *Staphylococcus aureus* were also screened for *MecA* gene.

### Antimicrobial drug susceptibility testing

Antibiotic susceptibility testing was performed using the disk diffusion method (Kirby Bauer) according to the CLSI criteria on Mueller-Hinton agar plates (OXOID). The antibiotics used in this study included vancomycin (30 µg), penicillin G (10iU), amoxicillin- clavulanic acid (20/10 µg), ofloxacin (5 µg), meropenem (10 µg), cefoxitin (30 µg), ceftazidime (30 µg), cefotaxime (30 µg), ciprofloxacin (5 µg), gentamicin (10 µg), erythromycin (15 µg), tetracycline (30 µg), chloramphenicol (30 µg), linezolid (30 µg) and trimethoprim sulfamethoxazole (1.25/23.75 µg). All the antibiotics were obtained from Oxoid laboratories (OXOID). Diameters of the zones of inhibition for individual antibacterial agents were translated into susceptible, intermediate, and resistant categories, according to the CLSI criteria [12]. Multi-drug resistant microorganisms were defined as resistant to at least three classes of antibacterial [12]. Isolates with zones of inhibition ≤ 27 mm for cefotaxime and ≤ 22 mm for ceftazidime were selected as potential ESBL producers.

### Preparation of DNA template for polymerase chain reaction

DNA extraction was undertaken using the Quick-DNA™ Miniprep plus kit (Inqaba Biotec West Africa Ltd) to extract and purify the bacteria genomic DNA according to manufacturer’s instructions. DNA concentrations and purity was determined using Nanodrop Spectrophotometer ND-1000 (USA) and read at 280 nm and extracted DNA was stored at − 20 °C.

### Genotyping of ESBL producing strains

Genotyping of ESBL producing strains for beta-lactamase genes TEM, SHV, CTX-M and VEB was performed as described by Trung et al. [13]. The multiplex PCR was optimized according to the following experimental conditions: Thermal cycling comprised initial denaturation at 95 °C for 4 min, 35 cycles of 94 °C for 25 s, 58 °C for 45 s and 72 °C for one minute [13].

### Detection of MecA Gene by Polymerase Chain Reaction (PCR).

The extracted DNA from *Staphylococcus aureus* was subjected to PCR for detection of *MecA* gene using the primer supplied by Inqaba biotec (see Table 1). Cycling parameter consists of initial denaturation at 94 °C for 30 min, denaturation at 94 °C for 30 s, primer annealing at 55 °C for 30 s, extension at 72^o^C for 1 min and final extension at 72 °C for 10 min. For quality control, *Escherichia coli* ATCC 25,922, *S. aureus* ATCC 25,923, *S. aureus* ATCC 29,213 (*MecA* negative), and *S. aureus* ATCC 700,699 (*MecA* positive) were used.

**Table 1:** List of primers used for the study

Target	Primer	Product size (BP)	References
mecA	F: AAAATCGATGGTAAAGGTTGGC R: AGTTCTGCAGTACCGGATTTGC	532	[14]
TEM	F: TCGCCGCATACACTATTCTCAAGAATGAC R: CAGCAATAAACCAGCCAGCCGGAAG	422	[13]
SHV	F: TGTATTATCTC(C/T) CTGTTAGCC(A/G) CCCTG R: GCTCTGCTTTGTTATTCGGGCCAAGC	739	[13]
CTX-M	F: ATGTGCAGYACCAGTAARGTKATGGC R: GGTRAARTARGTSACCAGAAYCAGCGG	590	[13]
VEB	F: GATGGTGTTTGGTCGCATATCGCAAC R: CATCGCTGTTGGGGTTGCCCAATTTT	391	[13]

## Result

Four hundred and ninety-nine (499) patients consented and participated in this study consisting of 412 (82.6%) females and 87 (17.4%) males (Table 2). The mean age ± SD of participants was 33.9 ± 13.8 with a range of 1–89 years. The majority (90.8%) of the participants were outpatients. Two hundred and thirty-two (232) isolates were obtained from 219 samples comprising of 120 (51.7%) Gram positive and 112 (48.3%) Gram negative bacteria (Table 2). Key bacteria pathogens isolated from this study include *Staphylococcus aureus* (22.8%), *Staphylococcus spp* (15.9%), *Escherichia coli* (16.4%), *Enterococcus spp* (7.3%) and *Klebsiella pneumoniae* (6.5%) see Table 3. The highest number of isolates was obtained from MGH indicating a higher infection rate in this region (Table 4), however there was no significant difference in the distribution of multi-drug resistant bacteria across the four centres (X^2^ = 5.47, p = 0.49).

**Table 2:** Socio-demographics of participants

Variable	Number (%)
*Gender *	
Female	412 (82.6)
Male	87 (17.4)
Total	499 (100)
*Patient status*	
In-patient	45 (9)
Out-patient	453 (90.8)
Missing	1 (0.2)
*Age category Mean age ± SD = 33.86 ± 13.8 (1–89 years)*	
0–20	56 (11.2)
21–40	326 (65.3)
41–60	85 (17)
60 and above	27 (5.4)
Missing	5 (1)
*Healthcare facility*	
LIMH	140 (28.1)
MGH	143 (28.7)
RGH	125 (25.1)
SGH	91 (18.2)
*Specimen type*	
Urine	269 (53.9)
High Vaginal Swab	161 (32.3)
Wound swab	17 (3.4)
Stool	12 (2.4)
Semen	11 (2.2)
Sputum	10 (2)
Endocervical swab	9 (1.8)
Ear swab	4 (0.8)
Urethral swab	4 (0.8)
Throat swab	I (0.2)
Abdominal abscess	1 (0.2)
Total	499 (100)
*Gram reaction N = 232*	
Gram positive	120 (51.7)
Gram negative	112 (48.3)
Total	232

**Table 3:** Distribution of Multi-drug resistance (MDR) across the bacterial species. The WHO priority pathogens are highlighted in bold

Bacterial specie	No Isolated	Frequency of MDR Number (% of species identified)
*Staphylococcus aureus*	**53**	**44 (83)**
*Escherichia coli*	**38**	**32 (84.2)**
*Staphylococcus spp*	39	23 (58.9)
*Klebsiella pneumoniae*	**15**	**13 (86.7)**
*Citrobacter koseri*	12	10 (83.3)
*Enterobacter aerogenes*	12	7 (58.3)
*Enterococcus spp*	**17**	**16 (94.1)**
*Streptococcus spp*	**11**	**11 (100)**
*Klebsiella spp*	**8**	**8 (100)**
*Citrobacter freundii*	4	3 (75)
*Enterobacter cloacae*	4	1 (100)
*Acinectobacter baumannii*	**3**	**2 (66.7)**
*Pseudomonas aeruginosa*	**3**	**3 (100)**
*Burkholderia cepacian*	2	2 (100)
*Proteus mirabilis*	2	1 (50)
*Pseudomonas oryzihabitans*	2	2 (100)
*Salmonella spp*	**2**	**1 (50)**
*Chromobacterium violaceum*	1	1 (100)
*Cronobacter sakazaki*	1	1 (100)
*Micrococcus luteus*	1	1 (100)
*Pluralibacter gergoviae*	1	1 (100)
*Stenotrophomonas xanthoma*	1	1 (100)
Total	**232**	**184 (79.3)**

Bold signifies WHO priority pathogens

**Table 4:** Distribution of isolates across the centres

Sentinel centres	No of patients with request for culture within the study period	No of patients who consented and participated in the study	No of isolates obtained	MDR prevalence (%) X2 = 5.47 P = 0.49
LIMH	402	140 (34.8%)	36 (25.7%)	29 (80.6)
MGH	302	143(47.4%)	113 (79%)	88 (77.6)
RGH	330	125 (37.8%)	38 (30.4%)	29 (76.3)
SGH	691	91 (13.2%)	45 (49.5%)	38 (84.4)
Total	1725	499	232 (46.5%)	184 (79.3)

**Fig. 2:**
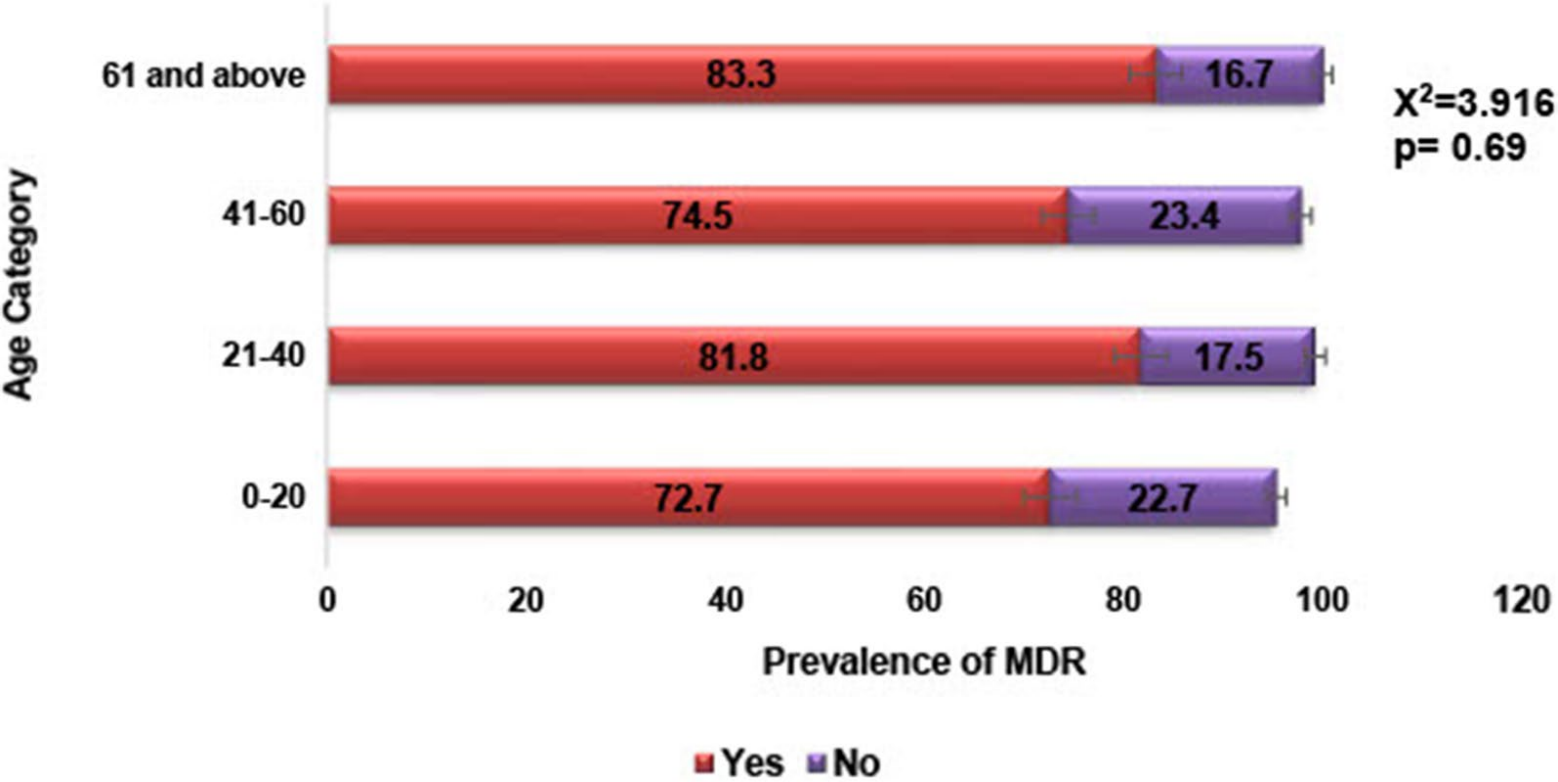
Distribution of Multi-drug resistance across the age categories

**Fig. 3:**
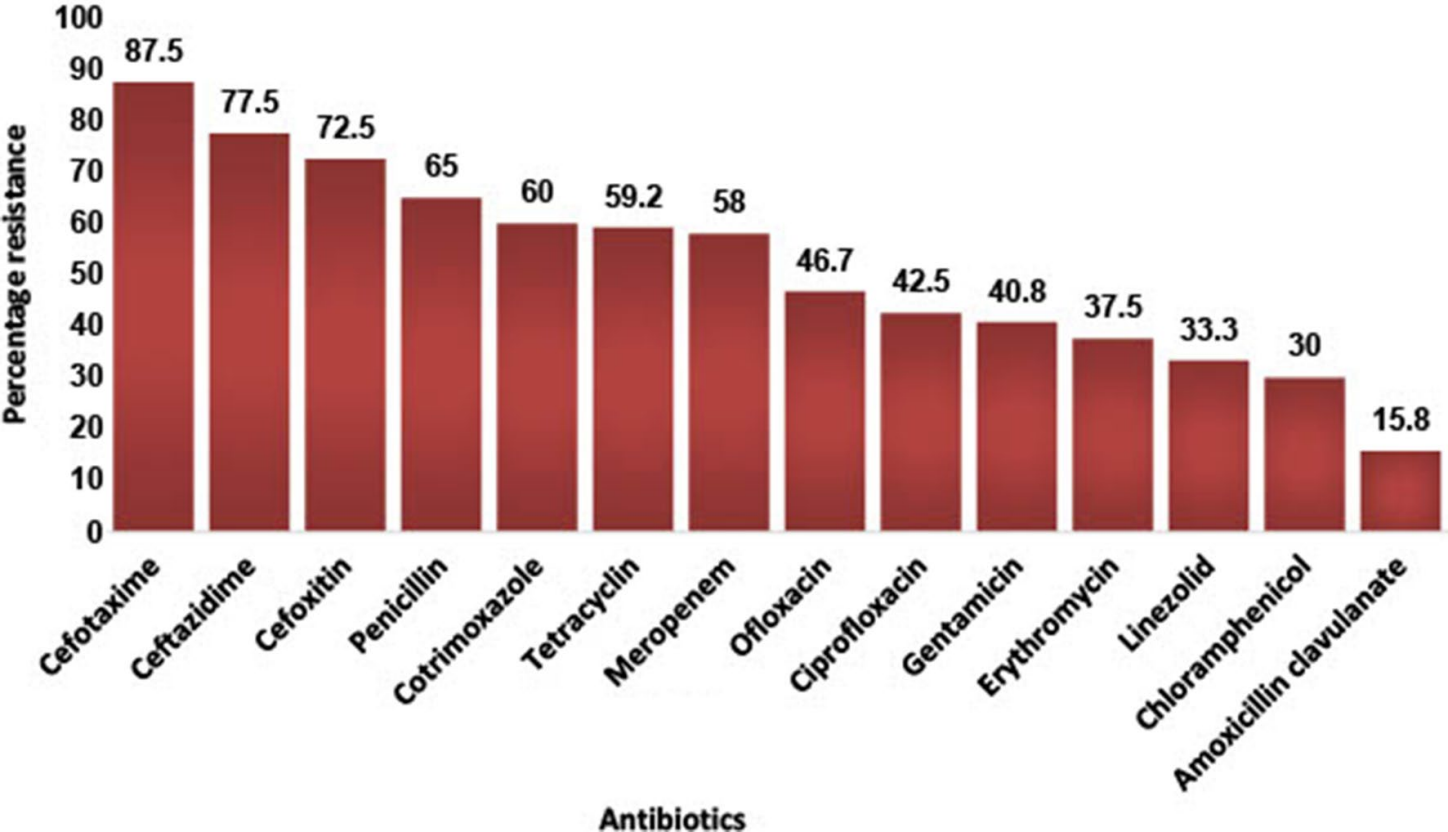
Resistance profile of Gram-positive bacteria isolates

**Fig. 4:**
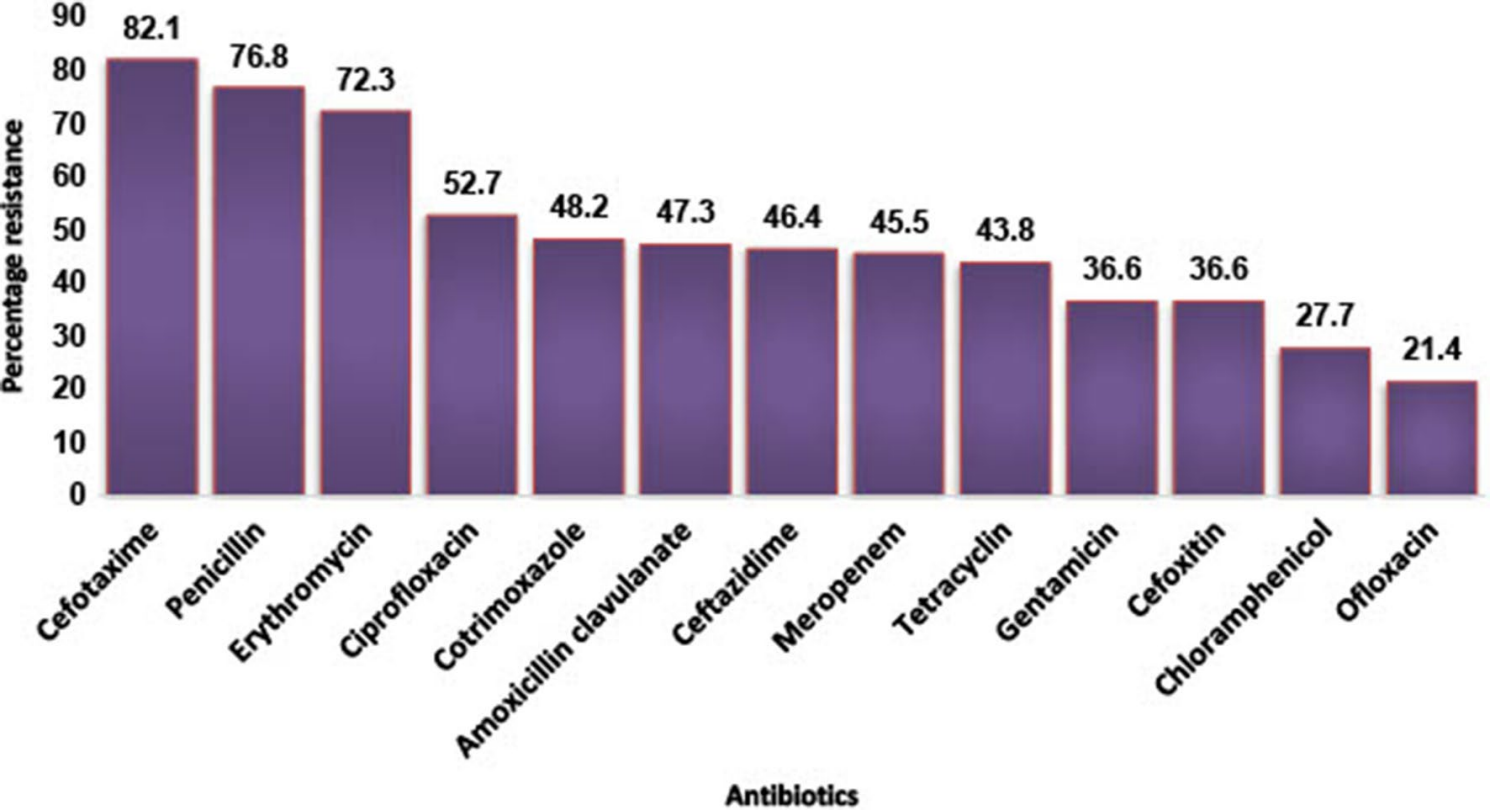
Resistance profile of Gram-Negative bacteria isolates

**Fig. 5:**
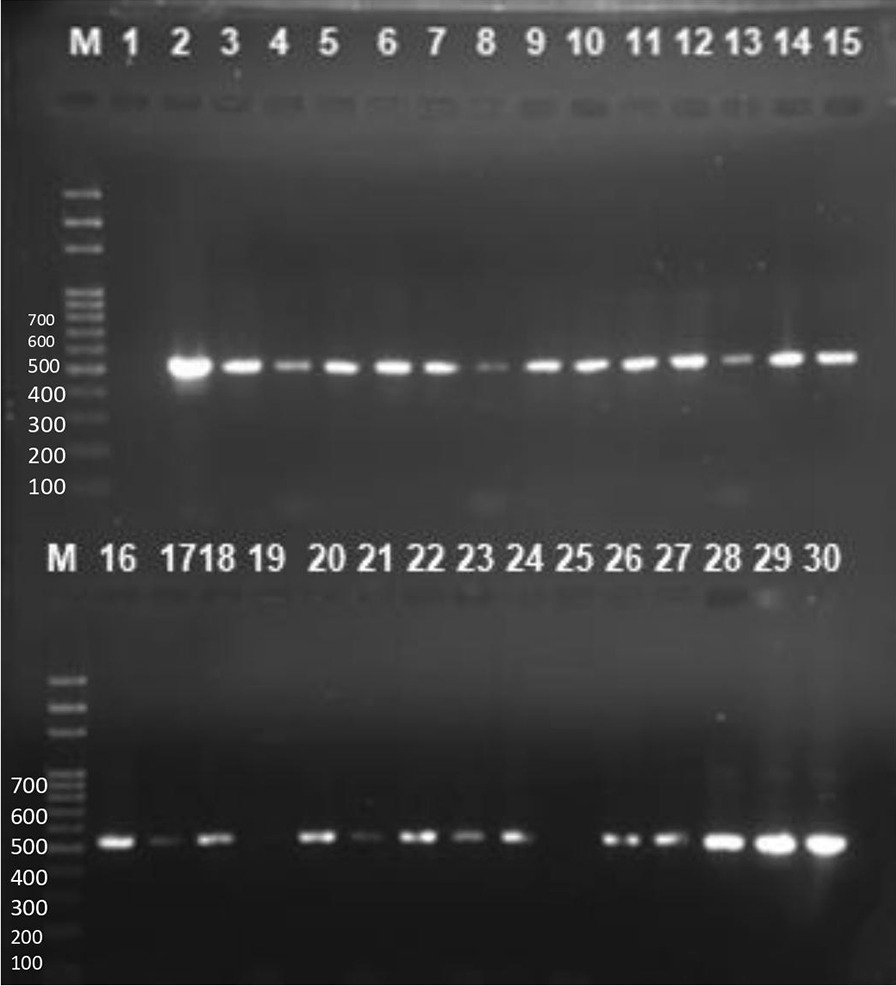
Agarose gel containing representative amplicon for the detection of MecA genes. Lane M, DNA ladder, Lane1 is a negative control, lane 2 is a positive control while Lane 3 to 30 contains representative band for MecA gene at 532 bp

There was high prevalence of multi-drug resistance (79.3%) among the isolates with *E. coli*, *S. aureus* and *K. pneumoniae* showing 84.2%, 83% and 86.7% resistance respectively (Table 3). Although the majority of the participants were within the age range of 21–40 years (Table 2), there was no significant difference in the distribution of multi-drug resistant bacteria across the age categories (X^2^ = 3.92, p = 0.69)—see Fig. 2. A few of the participants (6.9%) reported exposure to domestic animals with the majority being exposed to dogs (43.8%) and poultry (25.0%). Participants who were exposed to domestic animals were more likely to have multi-drug resistant infections (X^2^ = 7.963, p = 0.019). In general, resistance to Cephalosporins was highest (Figs. 3 and 4) while 76.5% of *Enterococcus spp* isolated were resistant to vancomycin. The majority (73.6%) of *Staphylococcus aureus* were phenotypically resistant to methicillin and 70% possessed the *MecA* gene (Fig. 5).

### ESBL genotyping using bla_CTX−M_, bla_TEM,_ bla_SHV_ and bla_VEB_

**Fig. 6:**
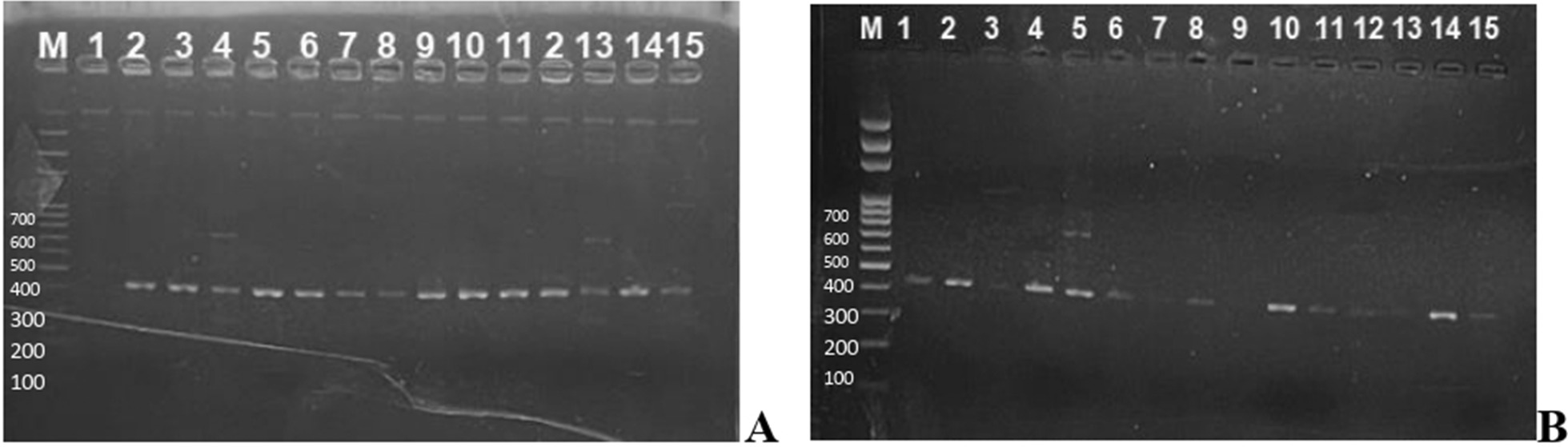
Agarose gel showing amplicon for bla_TEM,_ and bla_SHV_ ESBL genes with 422 bp and 739 bp band size respectively. Figure 6A lane 4 &13 and Fig. 6B lane 5 shows bands for bla_TEM,_ bla_SHV_ genes

Out of 57 Gram negative isolates subjected to ESBL genotyping (37 *E.coli* and 20 *Klebsilella spp*), 46 (80.7%) had at least one ESBL gene. Of these, 38(66.7%) had bla_TEM ,_ 3 (5.3%) had only bla_SHV_ while 5(8.8%) had both bla_TEM_ and bla_SHV_ genes. Eleven (19.3%) were negative to all the ESBL genes tested (Fig. 6). None of the isolates exhibited bla_CTX−M_ or bla_VEB_ ESBL genes.

## Discussion

Infections caused by multi-drug resistant bacteria are on the rise leading to treatment failure, prolonged hospital stay, and death [11]. We report the results of a laboratory survey of AMR in four sentinel sites (general hospitals) in Lagos State. General hospitals (GH) in Lagos State are secondary public healthcare centres which are slightly more equipped than the primary health centres but have fewer facilities than the teaching hospitals (Tertiary). These hospitals are community-based entities that admit all types of medical and surgical cases but concentrate on patients with acute illnesses needing relatively short-term care. There was a female preponderance in the study participants which was partly due to the fact that one of the facilities (LIMH) was specifically a mother-and-child hospital. That notwithstanding, more women consented to participate in the study than males. This female preponderance has also been reported in a laboratory-based surveillance of AMR in Ghana [15].

The facilities being general hospitals, had limited bedspace, hence the lower number of in-patient (9%) compared to out-patient (90.8%) participants. Also, the COVID-19 pandemic and partial lock down during the study period may have affected the number of in-patients as people were generally weary of going to hospitals during that period. Although the highest number of isolates were obtained from MGH showing a higher rate of infection in this region, there was no significant difference in the distribution of multi-drug resistant bacteria across the four centres (p = 0.49).

Gram-positive and gram-negative bacteria were identified in the current study, consistent with other surveillance studies [15–17]. Although some opinions have suggested the focus on WHO priority specimen and pathogens in surveillance, there are contrary opinion to this [18, 19]. This study considered all clinical specimens received for culture at the sentinel sites during the study period, and therefore, captured both priority and non-priority pathogens which may be contributing to AMR through transfer of antibiotic resistance genes. The most prevalent pathogens isolated in the current study included *Escherichia coli* and *Staphylococcus aureus* with *K. pneumoniae, E. coli* and *S. aureus* and showing the highest prevalence of multi-drug resistance, in 86.7%, 84.2% and 83% of clinical isolates respectively. These findings are similar to the report of surveillance studies conducted elsewhere in Ghana [15], Europe [20], and Southwestern Nigeria [21]. Exposure to domestic animals was a significant risk factor for multi-drug resistant infection in this study. The abuse of antibiotics in the care of domestic / companion animals including dogs, poultry and fish has been recognized as a major driver of AMR. Iramiot et al. [22] reported high prevalence of multi-drug resistance among organisms from both human (93%) and animal (83%) rectal swabs in Southwestern Uganda and suggested a high likelihood of transmission of multi-drug resistance between humans and animals. This reiterates the need for a one health approach towards understanding the true burden of MDR pathogens, especially in low-and-middle income countries.

The present study showed high prevalence of resistance to third-generation cephalosporins across both Gram-positive and Gram-negative bacteria isolated. Increasing trend of resistance to third generation cephalosporins have been reported by previous studies in Nigeria [23, 24]. This may likely indicate an overuse of these group of antibiotics. A point prevalence of survey to determine the rate of antibiotic prescription in four tertiary hospitals in Nigeria, reported that about 50% of prescriptions in these hospitals lacked clear therapeutic indications and third generation cephalosporins was fingered as the most prescribed antibiotics [25]. Third-generation cephalosporins are broad-spectrum antibiotics that are useful in the treatment of variety of clinical infections. Therefore, high levels of resistance to these antimicrobials are worrisome. Also, antibiotics of last resort, such as linezolid, a WHO reserve antibiotic [26] for the treatment of infection caused by multi-drug resistant Gram-positive bacteria, showed a concerning susceptibility profile with 33% resistance (Fig. 3). The high prevalence of MDR (79.3%) recorded in this study has also been reported elsewhere in Iraq with 75% and 87.5% MDR phenotypes for *K. pneumoniae* and *E. coli* isolates respectively [27]. This is a huge concern for clinical epidemiology and infectious diseases management.

Methicillin resistant *Staphylococcus aureus* (MRSA) is emerging as one of the major pathogens of public health concern. Methicillin resistance in *Staphylococcus aureus* confers resistance to the entire classes of -lactams including cephalosporins and carbapenems and has a higher risk of development of resistance to the quinolones, aminoglycosides, and macrolides [28, 29]. The high prevalence of MRSA (73.6%) recorded in this study is a source of major concern and this trend has also been reported in previous studies [30]. However, Adhikari et al. [31] reported a lower prevalence (35.5%) in *S. aureus* isolated from wound/pus of patients attending a tertiary care hospital in Kathmandu, Nepal. The *MecA* gene was not present in some of the strains of MRSA screened by cefoxitin disc diffusion method, but CLSI guidelines stipulates that *S. aureus* isolates should be regarded as MRSA if they are found resistant to either cefoxitin or oxacillin or both regardless of the presence or absence of *MecA* gene [32].

The use of multiplex PCR to screen for bla_CTX− M_, bla_TEM,_ bla_SHV and_ bla_VEB_ ESBL genes in *Escherichia coli* and *Klebsiella spps* from this study showed high prevalence (80.7%) of ESBL genes with 66.7% having bla_TEM_ gene. This is contrary to global picture in which the most common type of ESBL is reported to be CTX-M-type ESBLs when compared to SHV and TEM ESBLs [33]. Our study did not record any CTX-M genes among the tested isolates. High prevalence of bla_TEM_ has also been reported by Pishtiwan and Khadija [27] among ESBL-producing *Klebsiella pneumoniae* (81%) and *Escherichia coli* (64.7%) isolated from thalassemia patients in Erbil, Iraq. Similarly, Ghorbani-Dalini et al. [34], reported a higher prevalence of bla_TEM_ gene (83.33%) and concluded that the bla_TEM_ gene for ESBLs-producing *E. coli* was widespread in Iran.

ESBL production in certain bacteria strains can precipitate resistance to other classes of antibiotics (aminoglycosides, quinolones, and sulfonamides) complicating treatment strategies [35]. Multi-drug resistant ESBL producing bacteria was found to be generally high in this study. The higher prevalence of bla_TEM_ recorded in this study buttresses the need for periodic monitoring of resistance patterns and resistance genes of bacterial pathogens in a geographical area for adequate control and surveillance of antibiotic resistance.

The high level of antibiotic resistance recorded in this study has dire implication for empirical treatment. There is urgent need for development of suitable surveillance tools, especially for monitoring AMR to ensure periodic review/update of empirical treatment guideline. The strength of this surveillance model is that unlike the sentinel model with teaching hospitals proposed by Mohammed et al. [36] and currently being conducted by the Nigerian Center for Disease Control (NCDC), this model uses General hospitals which are usually the first point of call for citizens seeking healthcare and has the ability to include the grass root population. The high number of out-patients (90.8%) in this study invariably provides an insight on the community prevalence of AMR. Furthermore, teaching hospitals in Nigeria are referral centres where majority of the cases are chronic and have undergone initial treatment at the referring centres with little or no success. As a result, survey of general hospitals has the potential to provide a more realistic picture of the burden of AMR in the locality.

### Limitations

Some facilities had various challenges such as the breakdown of the autoclave and incubators plus stock outs which led to interruption in sample collection and processing. Also, COVID-19 pandemic and its attendant issues including partial to total lockdown drastically reduced the number of people presenting at the centres for microbiological tests during the study period.

Some participants declined consent to participate citing concerns that their samples were being used for COVID-19 research. Although efforts were made to educate the patients properly on the objectives of the study, a lot of people declined participation, and this greatly affected the uptake of study especially at the SGH site. Furthermore, the sites did not process specialized samples such as blood culture and cerebrospinal fluid (CSF) during the study period. It may either be that these tests are not routinely requested for, or they lacked adequate facilities for processing them. The study however, analysed routine samples collected at the designated centres and had no influence on the examination requested or the sample type. There was a female preponderance in the study which was partly due to the fact that one of the centres was a “mother and child” hospital (LIMH). However, the perception of general hospitals, as more of maternity centres may have also contributed to the higher number of females than males. Additionally, females are more likely to give their consent and participate in health studies than males who may perceive it as time wasting.

## Conclusion

Based on our findings in this pilot study, there was a high prevalence of AMR in clinical bacteria isolates from general hospitals in Lagos State stressing the need for urgent implementation of national action plans to tackle AMR. It is clear that laboratory-based AMR surveillance by isolate collection can yield reliable data on the resistant profile and possible trend in the emergence and spread of resistance. Sentinel protocols involving the collection of isolates may have the common drawback of producing a smaller dataset, but it has the advantage of high reproducibility since all isolates are tested under the same conditions using standardized methods and antibiotic panels. This is crucial in a resource poor setting where healthcare centres have limited facilities for monitoring AMR and further ensures uniformity of data generated for ease of comparability. We recommend that this approach be standardized and escalated for investigating and monitoring of AMR in other states with the aim to ascertain the burden of AMR in Nigeria.

## Supplementary Information

**Additional file 1.** Antimicrobial Resistance Surveillance, Checklist for evaluation of competency of study centers.

## Data Availability

All data generated or analysed during this study are included in this published article [and its supplementary information files] Original data set are available from the corresponding author upon reasonable request.

## Abbreviations

AMR: Antimicrobial resistance
ESBL: Extended spectrum beta-lactamase
CLSI: Clinical Laboratory Standard Institute
DNA: Deoxyribonucleic acid
NIMR: Nigerian Institute of Medical Research
SPSS: Statistical package for social sciences
GAP: Global action plan
NAP: National action plan
MDR: Multidrug resistance
LIMH: Lagos Island Maternity Hospital
MGH: Mushin General Hospital
RGH: Randle General Hospital
SGH: Shomolu General Hospital
