# Supplementary material for: High prevalence of resistance to third-generation cephalosporins detected among clinical isolates from sentinel healthcare facilities in Lagos, Nigeria
Source: Antimicrob Resist Infect Control. 2022 Nov 8;11:134. doi: 10.1186/s13756-022-01171-2 (PMC9640893; doi:10.1186/s13756-022-01171-2)
Supplement: Supplementary file 1 — Additional file 1. Antimicrobial Resistance Surveillance, Checklist for evaluation of competency of study centers. [file 13756_2022_1171_MOESM1_ESM.pdf]

Supplementary 1

Nigerian Institute of medical Research, Yaba, Lagos.

Microbiology Department

Antimicrobial Resistance Surveillance, Checklist for evaluation of competency of study centres

Name of facility…………………………………………………………

Date:………………………………………………………..

S/N	Variable	Availability	Qualification/Functional Status	Number	Remarks
**1**	**Personnel**				
**2**	**Bacteriology Lab**				
**3**	**Culture bench**				
	**EQUIPMENT**				
**4**	–20°C Refrigerator				
**5**	–80°C Refrigerator				
**6**	Lyophilisation				
**7**	Scale or balance (specify type)				
**8**	Incubators				
**9**	Candle jars				
**10**	Anaerobe jar or cabinet				
**11**	CO_2 incubator				
**12**	CO_2 tanks				
**13**	Magnifying lens/colony counter				
**14**	Calibrated loops (0.01and 0.001ml)				
**15**	Bunsen burner or heater or lamp				
**16**	Petri dishes (glass / disposable)				
**17**	Staining facilities – sink and slide rack				
**18**	Adequate glassware for media preparation				
**19**	pH paper / pH meter				
**20**	Water distillation system				
**21**	Low-speed centrifuge				
**22**	Autoclave				
**23**	Hot air oven				
**24**	Water bath				
**25**	Light microscope				

**26**	McFarland standards				
**27**	Millimetre rulers or callipers				
**28**	Antibiotic disc dispenser				
	**RECORDS**				
**29**	Isolate ID records				
**30**	Susceptibility profile Records				
